# Cancer, stem cell misplacement and cancer stem cells

**DOI:** 10.1111/jcmm.12104

**Published:** 2013-07-16

**Authors:** Yong Liao

**Affiliations:** Institute of Hepatitis and Key Laboratory of Molecular Biology for Infectious Diseases Ministry of Education Center of Liver Diseases The Second Affiliated Hospital, Chongqing Medical UniversityChongqing, China

## Abstract

The cell of origin of cancer as well as cancer stem cells is still a mystery. In a recent issue of JCMM, Wang *et al*. challenged the conventional somatic genetic mutation model of multi-stage carcinogenesis of breast cancer and proposed that ‘Invasive cancers are not necessary from preformed *in situ* tumours—an alternative way of carcinogenesis from misplaced stem cells’. If this stem cell misplacement theory could withstand future experimental evaluation, it may provide a paradigm shift in the prevention and management of cancer in the clinic.

The cell of origin of cancer as well as cancer stem cells is still a mystery and under debate. Recent lineage-tracing experiments performed in a panel of solid tumours demonstrate multiple cells of origin of epithelial cancers, including a stem cell or progenitor origin of cancer [1, 2]. If an adult tissue stem cell or a progenitor cell is the cellular origin of cancer, a logic question is what had happened to that particular stem/progenitor cell before it can be the cell of origin of cancer? The lineage-tracing experiments were performed in animals with pre-defined genetic background with specific genetic mutations and thus, these model systems will not be able to address the situations associated with the spontaneous tumours developed in patients. According to the conventional models, such as the somatic gene mutation theory, the current prevalent theory of carcinogenesis, a somatic cell in the adult organism would undergo successive DNA mutations that enable the cell to evolve and acquire the malignant phenotypes [3, 4]. A hypothetic multi-stage model of breast carcinogenesis depicts that a normal mammary epithelial cells has to go, step-by-step, through a processes of multi-stage carcinogenesis—from hyperplasia to atypical ductal hyperplasia, to ductal carcinoma *in situ* (DCIS), to invasive carcinoma and metastatic carcinoma, in order to accumulate required somatic mutation events for the malignant phenotypes [5].

In the current issue of JCMM, Wang *et al*. challenged the conventional model of multi-stage carcinogenesis of breast cancer and proposed that ‘The invasive cancers do not all come from the *in situ* carcinomas’, based on their analysis of Her-2 overexpression in achieved tissue specimen and, inconsistent incidence between DCIS and mammary tumours [6]. In addition, Wang *et al*. challenged the current paradigm on the cellular origin of cancer-initiating cells and boldly proposed that ‘The true cancer develops *de novo* from a misplaced epithelial stem cell (EpSC)’, even though the identity of the EpSC is not clear and the mechanisms underlying EpSC misplacement are elusive.

If one analysis carefully of the hallmarks of cancer cells summarized by Hanahan and Weinberg [7], one would recognize that most of these hallmarks of cancer cells are also phenotypic traits of a stem cell. Although these traits might be gained, step-by-step, by differentiated somatic cells *via* dedifferentiation and/or accumulation of multi-stage mutational events, yet it could also be likely descended from their original ancestry, *i.e*. a stem cell.

In a review of literature, there are quite a few reports that are in support of the stem cell misplacement as a potential cause in the development of cancer. A teratoma—an encaposulated tumour with tissue or organ components resembling normal derivatives of all three germ layers—may be a good example in support of the stem cell misplacement theory (SCMT) [8, 9]. Formation of teratoma by subcutaneous inoculation of human embryonic stem cells (hESCs) or induced pluripotent stem cells (iPSCs) is now a widely used assay in determination of pluripotent properties of hESC or iPSCs [9]. Recently, there is a solid experimental evidence to demonstrate the bone marrow–derived stem cells as the cell of origin of Helicobacter-induced gastric preneoplasia and gastric cancer in animal models [10]. In addition, genetic lineage analyses of tumour cell origin of epithelial malignant tumours that developed after allogeneic bone marrow transplantation demonstrate the secondary cancers of the donor origin in the recipients [11]. Thus, these reports, in a way, support Wang's ‘stem cell misplacement’ notion. The SCMT also has certain similarities to ‘the tissue organization field theory’ of carcinogenesis proposed a decade ago by Sonnenschein and Soto [3]. However, there are quite a lot unanswered questions with regard to the current SCMT.

First of all, if mutation is not essential to carcinogenesis, what is the driving force that initiated EpSC misplacement into the stroma and EpSC carcinogenesis? A likely event might be tissue damage that could directly break the basement membrane, or tissue repair and reorganization during chronic inflammation and infection, which are the common cause of human cancers [2].

Second, if invasive carcinomas were not developed from DCIS, but resulted from misplacement of EpSC to the stroma, what kind of EpSC may give rise to a Her2-positive, an ER-positive or a basal phenotype breast cancer respectively? Obviously, the hypothetic models proposed by Visvader and Polyak depicting that different sub-types of breast cancers may originate from different breast progenitor cells and/or differentiated cells, should be far more acceptable than the current hypothesis in view of the cell origin of different sub-types of breast cancer [5, 12]. In addition, lineage-tracing experiments have validated that luminal progenitors induced basal-like breast cancer flowing BRCA1 and p53 deletion [1].

Moreover, it is quite intriguing why the misplacement has to be normal ‘stem cells’ but not progenitors or differentiated epithelial cells with or without genetic mutations? The histologist Charles Leblond described earlier in 1950s that there are three main mechanisms by which adult organs are maintained: static (no replication occurs, *e.g*. nervous system); self-renewal by stem cells; and simple duplication by proliferation of their own differentiated cells [2]. Currently, there are also accumulating evidence in support of differentiated cells, in addition to stem or progenitor cells as the cell of origin of cancer [1, 2]. Even though the ‘cancer stem cell hypothesis’ depicted a potential stem cell origin of cancer, it also posits that the ‘cancer stem cell’ may not necessary to be originated from a stem cell, it may just manifest, phenotypically, the stem cell properties, such as immortality and capacity of self-renewal of more differentiated daughter cells and the stem cell itself [2].

Cancer has also been described as ‘wounds that do not heal’, suggesting that it is likely that misplaced stem cell (from either haematopoietic or tissue origin) or progenitor cell during repeated tissue damage and repair processes (*e.g*. chronic inflammation and infection) may become a potential source of cell of origin of cancer. If this SCMT could withstand future experimental evaluation, it may not only improve our understanding of carcinogenesis, particularly the cellular origin of cancer and cancer stem cells, but also provide a paradigm shift in the prevention and management of cancer in the clinic.
